# An implantable loop recorder or smartphone based single-lead electrocardiogram to detect arrhythmia in adults with congenital heart disease?

**DOI:** 10.3389/fcvm.2022.1099014

**Published:** 2023-01-06

**Authors:** Maarten A. Koole, Dirkjan Kauw, Kirsten M. Kooiman, Joris R. de Groot, Danielle Robbers-Visser, Igor I. Tulevski, Barbara J. Mulder, Berto J. Bouma, Mark J. Schuuring

**Affiliations:** ^1^Department of Cardiology, Amsterdam UMC, University of Amsterdam, Amsterdam, Netherlands; ^2^Cardiology Centers of the Netherlands, Amsterdam, Netherlands; ^3^Department of Cardiology, Rode Kruis Ziekenhuis Beverwijk, Beverwijk, Netherlands; ^4^Department of Cardiology, Haga Teaching Hospital, The Hague, Netherlands; ^5^Netherlands Heart Institute, Utrecht, Netherlands; ^6^Department of Cardiology, UMC Utrecht, Utrecht, Netherlands

**Keywords:** arrhythmias, congenital heart disease, electrocardiography, telemedicine, implantable loop recorder, cardiology, pacemaker, eHealth

## Abstract

**Background:**

The European Society of Cardiology (ESC) guidelines for the management of adult congenital heart disease (ACHD) recommend screening in patients at risk for arrhythmic events. However, the optimal mode of detection is unknown.

**Methods:**

Baseline and follow-up data of symptomatic ACHD patients who received an implantable loop recorder (ILR) or who participated in a smartphone based single-lead electrocardiogram study were collected. The primary endpoint was time to first detected arrhythmia.

**Results:**

In total 116 ACHD patients (mean age 42 years, 44% male) were studied. The ILR group (*n* = 23) differed from the smartphone based single-lead electrocardiogram group (*n* = 93) in having a greater part of males and had more severe CHD and (near) syncope as qualifying diagnosis. In the smartphone based single-lead electrocardiogram group history of arrhythmia and palpitations were more frequent (all *p* < 0.05). Monitoring was performed for 40 and 79 patient-years for the ILR- and smartphone based single-lead electrocardiogram group, respectively. Arrhythmias occurred in 33 patients with an equal median time for both groups to first arrhythmia of 3 months (HR of 0.7, *p* = 0.81). Furthermore, atrial fibrillation occurred most often (*n* = 16) and common therapy changes included medication changes (*n* = 7) and implantation of pacemaker or Implantable Cardioverter Defibrillator (ICD) (*N* = 4). Symptoms or mode of detection were not a determinant of the first event.

**Conclusion:**

Non-invasive smartphone based single-lead electrocardiogram monitoring could be an acceptable alternative for ILR implantation in detecting arrhythmia in symptomatic ACHD patients in respect to diagnostic yield, safety and management decisions, especially in those without syncope.

## 1. Introduction

### 1.1. Adult congenital heart disease

Congenital heart disease has a worldwide prevalence of ∼9 per 1000 newborns. Nowadays, the number of adult congenital heart disease (ACHD) patients exceeds the number of children with congenital heart disease and the population of ACHD patients is still increasing by 5% per year (1, 2). These ACHD patients are under lifelong surveillance in specialized centers. Although their prognosis has significantly improved compared to only a few decades ago, these patients are not cured. Data from the Dutch National CONCOR registry showed that the median age of death is 49 years and that two third of adult patients with CHD die from a cardiac cause (3–6). One of the most common causes of death is sudden cardiac death (19%), which occurs at a median age of 39 years (3, 4, 7). It is estimated that 1 out of 6 ACHD patients develops bradycardias or tachyarrhythmia during life, that often precede syncope and/or sudden death (3). Over one-third of tetralogy of Fallot (ToF) patients develop symptomatic atrial tachyarrhythmia by adulthood, 10% develop high-grade ventricular arrhythmia, and 5% require a pacemaker implantation for surgically acquired atrioventricular block or sinus node dysfunction. After Senning or Mustard repairs for Transposition of the Great Arteries (TGA), loss of sinus rhythm occurs in 60% of patients in the 20-year period after surgery (8).

### 1.2. Arrhythmia detection

The European Society of Cardiology (ESC) guidelines recommend periodical screening in symptomatic ACHD patients, without arrhythmia documentation at presentation, evaluation for arrhythmia (1). Subgroups of patients who are at increased risk are identified in the guideline. In patients with pacemakers or implantable cardioverter defibrillators (ICDs), device interrogation is used to screen for arrhythmias (9, 10). In patients without implantable device, short term screening is commonly performed with Holter studies, and prolonged screening with Implantable Loop Recorders (ILR). However, smartphone based single-lead electrocardiogram solutions may provide new alternatives (11, 12). Mobile devices for heart rhythm monitoring, defined as ambulant diagnostics, is rapidly evolving as wearables, mobile health applications (apps) and smartphone possibilities are improving, and increasing in number (13–15). ACHD patients seem particularly eligible to benefit from these alternative solutions, as these patients have a higher burden of arrhythmia compared to the general population and having their first arrhythmia at younger age. So they are generally well motivated to apply eHealth. However, data on smartphone based single-lead electrocardiogram are scarce. Therefore, the study aimed to explore whether smartphone based single-lead electrocardiogram can be a good alternative to ILR in detecting arrhythmia.

## 2. Methods

### 2.1. Study data

Baseline and follow-up data were collected of two cohorts of ACHD patients with symptoms which could be caused by arrhythmia. One cohort were patients who participated in a smartphone based single-lead electrocardiogram study and the other cohort are patients gathered by a retrospective chart review of patients with an ILR. Indications for ILR implantation were symptoms which could be related to arrhythmia. The smartphone based single-lead electrocardiogram group of patients participated in a prospective study in two medical centers in the Netherlands (Haga Teaching Hospital and Amsterdam UMC, location AMC). The study protocol required routine evaluation of heart rhythm using a wireless pocket-sized single lead EKG recording device that could record a 30 s single lead EKG (Kardia, AliveCor). After a 1-week run-in period, a single lead EKG was recorded once every week. Patients could perform extra measurements in case of symptoms. Data of events were sent by the application of the smartphone to our telemedicine center and within 48 h judged by specialized nurses. Data of the ILR were read as soon as possible after an event at our outpatient clinic. All patients were explained to contact a physician directly in case of emergency. Detailed description of the study has been published elsewhere (15). A retrospective chart review has been performed to collect ILR data of all symptomatic ACHD patients having an ILR implanted between 2003 and 2019 (Amsterdam UMC, location AMC).

### 2.2. Study criteria

The smartphone based single-lead electrocardiogram study ACHD patients were eligible for inclusion if they met the following inclusion criteria: palpitations within the last 3 years (with or without arrhythmia diagnosis) or HF NYHA class ≥ II, and possession of a smartphone. Patients with impaired cognition, as assessed by their treating physician, tremors or patients with an insurance not covering costs of the smartphone based single-lead electrocardiogram program, were excluded. Patients were recruited from the outpatient clinic and clinical wards. Enrollment in this study followed after informed consent for the use of their clinical data was acquired. The local medical ethics committees of both institutions issued a waiver for this study. This included a waived consent for the retrospective chart review, because data were processed anonymously by the investigator.

### 2.3. Study outcome

The primary endpoint was time to first arrhythmia detected (AF, SVT, VT, sinus node defect, or AV block) in both study groups. Device implantation and change in medication were not an outcome but also registered as a result of detecting arrhythmia for both groups. Data were analyzed with Kaplan-Meier survival curves and Cox proportional hazard analysis (SPSS version 28, IBM, Armonk, New York, NY, USA). Chi-square test or independent *t*-test were used to assess differences between patient-groups.

## 3. Results

### 3.1. Baseline characteristics

In total 116 ACHD patients were studied, see Table 1. Mean age was 42 years and 44% were male. There were 25 (22%) patients with mild CHD, 45 (39%) patients with moderate CHD, and 46 (39%) patients with severe CHD. The rate of hypertension (*n* = 16, 14%) or coronary artery disease was low (*n* = 7, 6%). The ILR group consisted of 23 patients and the smartphone based single-lead electrocardiogram consisted of 93 patients.

**TABLE 1:** Baseline characteristics.

	All	ILR	Smartphone ECG	*p*
	*N* = 116	*N* = 23	*N* = 93	
Age, years	42	44	42	0.573
Male, *N* (%)	51 (44)	17 (74)	34 (37)	**0**.**001**
**Severity of CHD**				
Mild, *N* (%)	25 (22)	3 (23)	22 (24)	**0**. **020**
Moderate, *N* (%)	45 (39)	5 (22)	40 (93)	
Severe, *N* (%)	46 (39)	15 (65)	31 (33)	
**Medical history**				
Cardiac surgery, *N* (%)	92 (79)	17 (74)	75 (81)	0.475
Non-cardiac surgery, *N* (%)	54 (47)	5 (22)	49 (53)	**0**.**007**
Coronary artery disease, *N* (%)	7 (6)	2 (9)	5 (5)	0.559
Arrhythmia, *N* (%)	91 (78)	10 (43)	81 (87)	**< 0**.**01**
Heart failure, *N* (%)	22 (19)	3 (13)	19 (20)	0.418
Hypertension, *N* (%)	16 (14)	1 (4)	15 (16)	0.142
Systemic EF < 40%, *N* (%)	6 (5)	0	6 (6)	0.208
Subpulmonic EF < 40%, *N* (%)	4 (3)	1 (4)	3 (3)	0.399
**NYHA class**				
I, *N* (%)	90 (78)	16 (70)	74 (80)	0.303
≥ 2, *N* (%)	26 (22)	7 (30)	19 (20)	
Arrhythmia symptoms, *N* (%)	95 (82)	19 (83)	76 (82)	0.921
Palpitations, *N* (%)	78 (67)	8 (35)	70 (75)	**< 0**.**01**
Dyspnea, *N* (%)	12 (10)	2 (9)	10 (11)	0.772
(Near) syncope, *N* (%)	18 (16)	15 (65)	3 (3)	**< 0**.**01**
**Medication**				
Antiarrhythmic agents, *N* (%)	52 (45)	7 (30)	45 (48)	0.121
Diuretics, *N* (%)	13 (11)	3 (13)	10 (11)	0.701
Anticoagulation, *N* (%)	45 (39)	7 (30)	38 (41)	0.358

N, number; EF, ejection fraction; ILR, implantable loop recorder; CHD, congenital heart disease; NYHA, New York heart association. Bold values represent the significant values.

The ILR group (*n* = 23) differed from the smartphone based single-lead electrocardiogram group (*n* = 93) in having a greater part of males. They had more severe CHD and (near) syncope (65 vs. 3%) as qualifying symptom of possible arrhythmia. In the smartphone based single-lead electrocardiogram group history of arrhythmia and suffering from palpitations were more frequent.

### 3.2. Monitoring details

**FIGURE 1:**
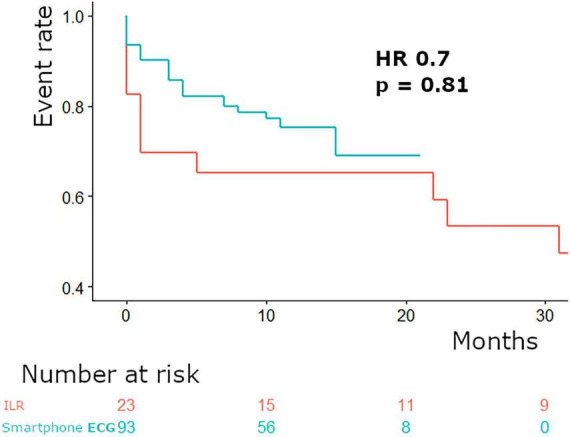
Time to first arrhythmia.

In total patients were monitored for 119 patient years. Monitoring was performed for 40 and 79 patient years, respectively, in the ILR and smartphone based single-lead electrocardiogram groups. The median time to first arrhythmia was 92 (16–233) days for the complete study cohort, for the ILR group 40 (15–681) days and for the smartphone based single-lead electrocardiogram group 102 (21–232) days (*p* = 0.80, HR of 0.7) (Figure 1 and Table 2). Arrhythmias occurred in 33 patients, of which 11 (48%) were documented in the ILR group and 22 (24%) in the smartphone based single-lead electrocardiogram group (*p* = 0.021). In both groups atrial fibrillation was the most frequently documented arrhythmia and no patient died.

**TABLE 2:** Details on monitoring.

A				
	All	ILR	Smartphone ECG	*p*
	*N* = 116	*N* = 23	*N* = 93	
Median time to first arrhythmia, days (IQR)	92 (16–233)	40 (15–681)	102 (21–232)	0.801
Median monitoring time per patient, days (IQR)	322 (148–428)	567 (40–1217)	317 (188–399)	**0.045**
**B**				
**Details on first arrhythmia**				
	**All**	**ILR**	**Smartphone**	***p***
	***N*** **= 116**	***N*** **= 23**	***N*** **= 93**	
Arrhythmia occured, *N* (%)	33 (28)	11 (48)	22 (24)	**0.021**
Atrial fibrillation, *N* (%)	16 (14)	2 (9)	14 (15)	0.428
Supraventricular tachycardia, *N* (%)	14 (12)	6 (26)	8 (9)	**0.021**
Ventricular tachycardia, *N* (%)	1 (1)	1 (4)	0	**0.043**
Sinus node defect, *N* (%)	2 (2)	2 (9)	0	**0.004**
Atrioventricular block, *N* (%)	0	0	0	1.000

N, number; IQR, interquartile ranges; ILR, implantable loop recorder. Bold values represent the significant values.

### 3.3. Changes in patient management

**FIGURE 2:**
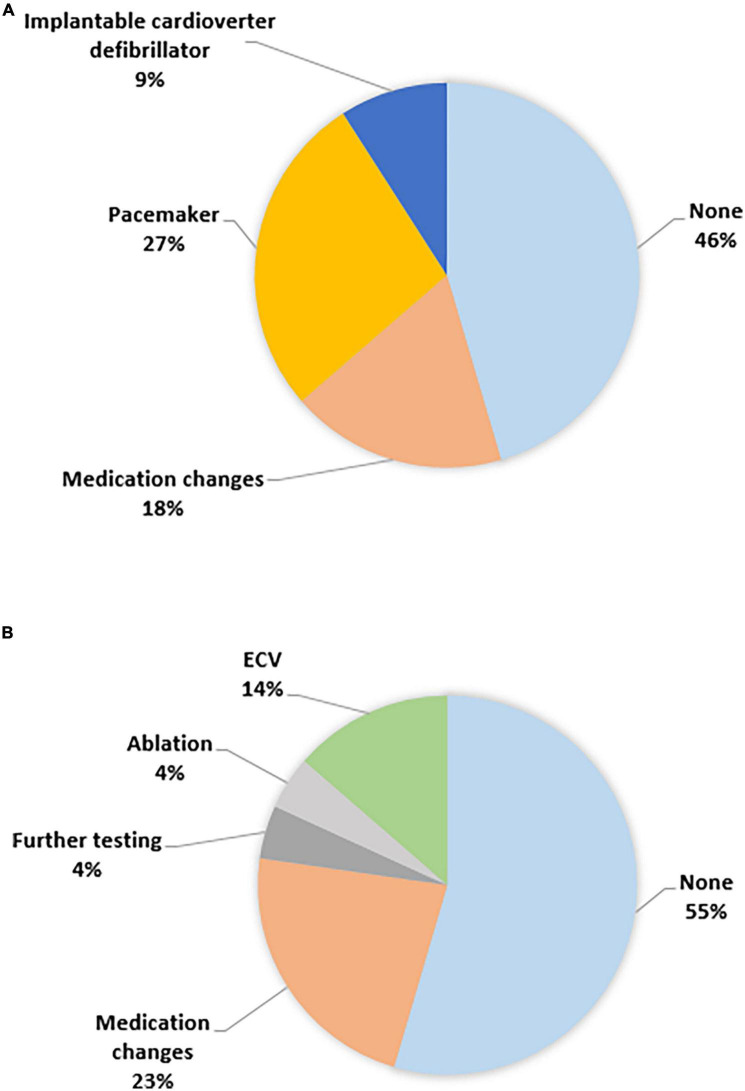
Care changes **(A)** ILR and **(B)** smartphone ECG.

Arrhythmia detection led to the important care changes, displayed in Figure 2. In the ILR group device implantation to treat arrhythmia was performed in four patients (three pacemaker and one ICD) and medication changes were performed in two patients (start of beta-blocker). Furthermore, in the ILR group a wait and see strategy was chosen in five patients. In the smartphone based single-lead electrocardiogram group ablation was performed in one patient and electrical cardioversion was performed in three patients. In five patients monitored with smartphone based single-lead electrocardiogram medication changes were performed, including start of a direct oral anticoagulant, start of amiodarone, and both start and increase of beta blocker. In one patient it was decided to perform additional Holter monitoring and in 12 patients no change in management was initiated.

### 3.4. Determinants of the first arrhythmia event

The mode of detection (HR 0,688 95% CI 0.3–1.6, 0,371) appeared not to be associated with the first detection of arrhythmia in the study period (HR 3.2, 95% CI 1.5–6.8, *p* = 0.002). The use of anti-arrhythmic drugs was associated with an arrhythmia event because patients with anti-arrhythmic drugs are at high risk of arrhythmia.

## 4. Discussion

### 4.1. Principal findings

Rhythm monitoring is important in ACHD patients as they are at high risk for arrhythmic and brady-arrhythmic events, but with the currently expanded possibilities of diagnostics no optimal diagnostic strategy has been defined yet. To our knowledge this is the first study that performed a comparison of ILR and smartphone based single-lead electrocardiogram for heart rhythm monitoring in ACHD patients. Smartphone based single-lead electrocardiogram seems to be a reasonable non-invasive alternative diagnostic tool for symptomatic patients instead of an invasive ILR for detecting arrhythmia.

### 4.2. Diagnostic yield of ambulatory rhythm monitoring in ACHD patients

Our findings of a high burden of arrhythmia in selected ACHD patients is comparable to the literature. Dodeja et al. evaluated traditional ILR monitoring in ACHD patients and showed a useful adjunct with clinically relevant events in 41% of patients (9). Schultz et al. performed a retrospective cohort study on remote ambulatory monitoring in 307 ACHD patients with symptoms, a history of arrhythmia or screening due to an increased risk. Their 14-day screening detected arrhythmia in 153 (50%) ACHD patients. Management changes, including medication changes (30%), further testing or imaging (10%), and procedures (6%), were made based on results of these prolonged monitoring strategy (16). Huntgeburth et al. performed a single center, retrospective observational study in which all CHD-patients with an ILR who were under care of the German Heart Center Munich between February 2015 and January 2019 were identified (17). The authors found a considerable complementary diagnostic value of ILR for the detection and differentiation of benign and malignant arrhythmias. Huntgeburth et al. concluded that ILR implantation should be considered in patients with CHD of any complexity who need medium or long-term arrhythmia monitoring, especially if short-term Holter monitoring cannot provide sufficient diagnostic certainty.

### 4.3. Smartphone based single-lead electrocardiogram for heart rhythm monitoring in ACHD patients

Smartphone based single-lead electrocardiogram is a promising tool to improve care and detect arrhythmia in ACHD patients (18–21). Smartphone based single-lead electrocardiogram has been shown to enable early detection of recurrences and new diagnosis of arrhythmia, which led to swift therapeutic response or remote reassurance. Furthermore, smartphone based single-lead electrocardiogram was well accepted in ACHD patients with high adherence and positive patient experience (15, 22). The risk of ILR implantation such as need for re-implantation, wound dehiscence or device erosion of 1–9% can be avoided (17, 23, 24). Smartphone based single-lead electrocardiogram as a non-invasive diagnostic tool has no such risk of surgical complications. In our analysis smartphone based single-lead electrocardiogram proofed to be an effective tool in detecting arrhythmia. In our study there was a lower rate of arrhythmia detection in the smartphone based single-lead electrocardiogram group, potentially due to the fact that this group had less patients with severe ACHD. Although ILR is better at detecting arrhythmias in patients because the window of measurement is continuous, it has the before mentioned disadvantage of being an invasive tool. So, we suggest in symptomatic patients, if symptoms occur on daily basis, 24 Holter monitoring for diagnosing arrhythmia is a good option. If symptoms occur less frequently smartphone based single-lead electrocardiogram could be an alternative option and save the ILR for patients where no diagnosis could be found with these modalities and for whom detecting arrhythmia is important to their prognosis. Furthermore, new wearables with smart algorithms can monitor patients continuous and alert patient and physician if arrhythmia is detected (25, 26).

### 4.4. Prolonged rhythm monitoring in acquired heart disease patients

Diagnostic yield of prolonged monitoring is also well established in AF screening in cryptogenic stroke patients (27). Longer durations of monitoring were associated with the highest diagnostic yield in these patients (28, 29). However, the optimal monitoring method and duration of monitoring is unclear (30–32). Solbiati et al. performed a systematic Cochrane review on ILR performance and concluded that available data are non-conclusive. The authors therefore recommended further research on ILR with clinically relevant outcomes (33). Our study suggests our smartphone based single-lead electrocardiogram protocol compared to ILR can be a good alternative in detecting arrhythmia in patients with symptoms other than syncope. Especially if these complaints are less frequent than once a day for which 24–48-h Holter monitoring is still a good alternative option.

### 4.5. Future directions

Beside clinical effectiveness other aspects of implementation include amongst others: cost evaluation, governance, patient, and technological factors. Studies on costs of smartphone based single-lead electrocardiogram are scarce. In the first study that compared eHealth with the standard outpatient clinic setting it was suggested that eHealth was likely cost-effective (34). That study was performed in patients who suffered from acute myocardial infarction. Hypothetically, smartphone based single-lead electrocardiogram is more cost-effective than ILR because it saves on the costs of implantation and explantation, but if wearables for heart rhythm monitoring use a service center with medical personnel, the costs for this solution could also become significant. Furthermore, health system governance, health provider, patient and technological factors may complicate implementation. However, tools to identify barriers to implementing digital health and recommendations for overcoming them are increasingly available (35–37).

### 4.6. Limitations

Our study was limited by a combination of two datasets, without randomization of patients between the two monitoring strategies. Moreover, short arrhythmia and asymptomatic arrhythmia or bradycardias may remain unnoticed in both groups. Despite we screened all ACHD patients visiting our outpatient clinic between 2003 and 2019 for having an ILR, the number of patients we found having an ILR was much smaller compared to the smartphone based single-lead electrocardiogram group. We postulate that the threshold for using an invasive diagnostic tool to find arrhythmia in symptomatic patients is higher compared to non-invasive Holter monitoring. The decision to implant an ILR to detect arrhythmia was most often reserved for ACHD patients with unexplained syncope or cerebral vascular accident after unsuccessful period of Holter monitoring. However, in the emerging field of non-invasive wearable heart rhythm monitoring solutions we are the first to report a comparison in this high-risk patient population. Matching was not performed in the study. The smartphone based single-lead electrocardiogram has a significantly higher number of patients with a history of previous arrhythmia. Previous arrhythmia could make arrhythmia recurrence more likely than no previous arrhythmia. However, arrhythmia could also make arrhythmia recurrence less likely because of the treatment with anti-arrhythmic drugs. Potentially this could have introduced bias the process of arrhythmia detection.

## 5. Conclusion

Non-invasive smartphone based single-lead electrocardiogram monitoring could be an acceptable alternative in detecting arrhythmia in symptomatic ACHD patients instead of an ILR in respect to diagnostic yield, safety and management decisions, especially in those patients without syncope.

## Data availability statement

The data underlying this article will be shared on reasonable request to the corresponding author.

## Ethics statement

The studies involving human participants were reviewed and approved by the Amsterdam UMC. Written informed consent for participation was not required for this study in accordance with the national legislation and the institutional requirements. Written informed consent was not obtained from the individual(s) for the publication of any potentially identifiable images or data included in this article.

## Author contributions

MK, BB, and MS drafted the manuscript, which was critically revised and edited by BM, DR-V, DK, IT, JG, and KK. All authors agree to be accountable for all aspects of the work.
